# Machine learning: A non-invasive prediction method for gastric cancer based on a survey of lifestyle behaviors

**DOI:** 10.3389/frai.2022.956385

**Published:** 2022-08-16

**Authors:** Siqing Jiang, Haojun Gao, Jiajin He, Jiaqi Shi, Yuling Tong, Jian Wu

**Affiliations:** ^1^Department of Public Health, Zhejiang University School of Medicine, Zhejiang University, Hangzhou, China; ^2^Real-Doctor Artificial Intelligence Research Center, Zhejiang University, Hangzhou, China; ^3^Department of General Practice/Health Management Center, School of Medicine, The Second Affiliated Hospital of Zhejiang University, Hangzhou, China

**Keywords:** gastric cancer, non-invasive, machine learning, behavioral lifestyles, retrospective study

## Abstract

Gastric cancer remains an enormous threat to human health. It is extremely significant to make a clear diagnosis and timely treatment of gastrointestinal tumors. The traditional diagnosis method (endoscope, surgery, and pathological tissue extraction) of gastric cancer is usually invasive, expensive, and time-consuming. The machine learning method is fast and low-cost, which breaks through the limitations of the traditional methods as we can apply the machine learning method to diagnose gastric cancer. This work aims to construct a cheap, non-invasive, rapid, and high-precision gastric cancer diagnostic model using personal behavioral lifestyles and non-invasive characteristics. A retrospective study was implemented on 3,630 participants. The developed models (extreme gradient boosting, decision tree, random forest, and logistic regression) were evaluated by cross-validation and the generalization ability in our test set. We found that the model developed using fingerprints based on the extreme gradient boosting (XGBoost) algorithm produced better results compared with the other models. The overall accuracy of which test set was 85.7%, AUC was 89.6%, sensitivity 78.7%, specificity 76.9%, and positive predictive values 73.8%, verifying that the proposed model has significant medical value and good application prospects.

## Introduction

Gastric cancer (GC) is a common malignancy arising from the epithelium of the gastric mucosa, with the third-highest mortality rate among the cancers worldwide (Bray et al., 2018), and approximately 478,508 new GC cases in China were confirmed in 2020 (Sung et al., 2021). In most cases, early-stage GC are asymptomatic, and a large proportion of patients are diagnosed at an advanced-stages (Correa, 2013). Unfortunately, the prognosis of advanced GC is poor as a result, with an average five-year survival rate of less than 25%. Suppose populations are screened early to identify those at risk of developing cancer or potential early patients. In that case, the incidence of GC can effectively be reduced, and the 5-year survival rate will be improved through appropriate intervention and prevention (Selgrad et al., 2010). Hence, it is increasingly urgent to seek a diagnosis method of non-invasive, cheap, and time-saving GC identification with high accuracy to achieve early GC prevention.

Currently, methods for diagnosing gastric cancer include traditional approaches based on the physician diagnosis and machine learning methods based on artificial intelligence (AI) (Niu et al., 2020). The former specifically includes endoscopy and histopathological examination (Karimi et al., 2014). Existing circulating biomarkers for GC diagnosis have low sensitivity, and GC diagnosis is only based on invasive procedures such as upper gastrointestinal endoscopy, in spite of the substantial diagnostic precision endoscopy can achieve, it may cause discomfort in patients through an invasive pathway (Leja and Linē, 2021), moreover, the cost-effectiveness of intervention is unreasonable in part of the western populations. The diagnosis of precancerous lesions or cancerous tissues in pathological section examination requires physicians to consume much time marking each region in proper order (Wu et al., 2021). In contrast, machine learning methods show more advantages than traditional methods, they can optimize the feature extraction process and achieve better classification performance and generalization ability (Münzenmayer et al., 2009; Meng et al., 2021). Despite multiple linear regression (Marill, 2004) and logistics regression are widely applied to medical statistics analysis of influence factors and traditional gastric cancer risk early warning. Nevertheless, these statistics analysis methods have limitations that cannot deal well with the nonlinear relationships in biological information. Whereas machine learning (Handelman et al., 2018) (ML) is equipped to coordinate the variance and deviation of original data since both the nonlinear relationship of biomedical knowledge and higher-order interactions between variables can be solved well (Deo, 2015).

To a great extent, the performance of machine learning depends on the rationality of included variables. Lifestyle behaviors such as diet, drinking, and smoking are considered as significant causes of GC and main targets for primary prevention (Katzke et al., 2015). The infection of Helicobacter pylori (H. pylori) is the leading cause of stomach-related diseases and considered as a primary risk factor for GC (Wang et al., 2014). In addition, previous studies have confirmed serum pepsinogen characteristics as early diagnostic characteristics with a substantial precision for gastric cancer (Bornschein et al., 2012; Yuan et al., 2020). These characteristics are non-invasive, simple, and available in the practical work, which can be used as a reliable clinical diagnosis basis for gastric cancer diagnostic. In this work, we collected 13 clinically relevant variables: potential gastric cancer influencing factors (first-degree relatives GC history, vegetable, smoking, etc.) and some non-invasive clinical characteristics (serum pepsinogen level, Hp infection, etc.).

The objectives of the present study were to evaluate and sought out the optimal ML algorithm to develop an efficient, low-cost, safe, and noninvasive diagnostic method for gastric cancer. Fortunately, this study is the first to use both lifestyle behavior information and noninvasive clinical characteristics to diagnose gastric cancer.

## Materials and methods

### Methods overview

**Figure 1:**
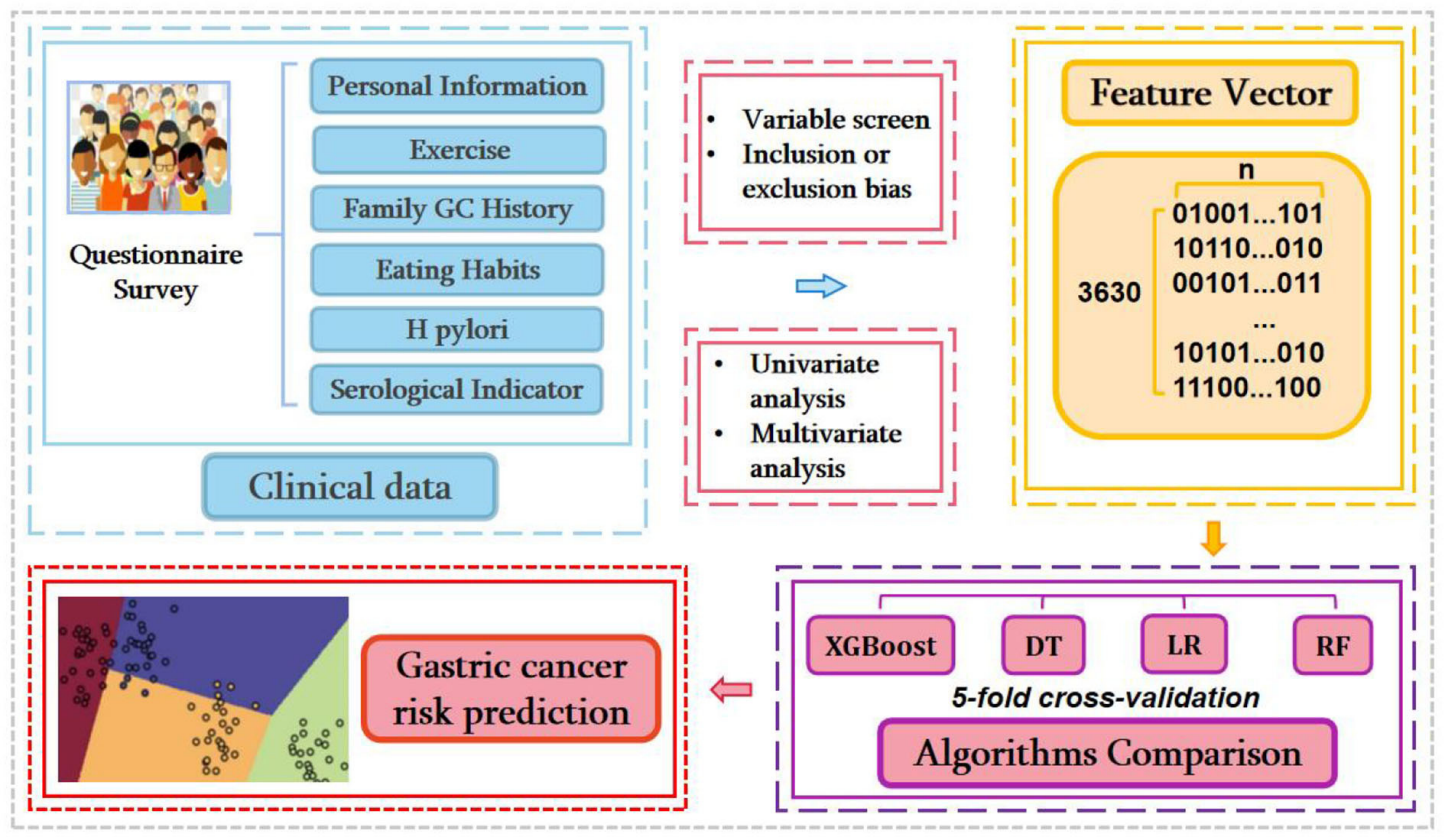
The workflow of current research.

This work mainly includes five sections: data collection, variable selection, feature extraction, model training, and intelligent prediction. The framework of our experiment procedure was showed in Figure 1. Information was collected by measuring subjects using questionnaires (self-reported). Investigators were demanded to uniform standards and questioning patterns and conducted special training on the questioning pattern and classification of qualitative characteristics.

The selection criteria of independent variables included in the machine learning model are as follows:

- Variables should be a potential GC influencing factor.
- Variables must possess the exact definition and use the same measurement methods throughout the experiment and investigation.

According to the criterion mentioned earlier, 13 variables were eligible for further study (Table 1).

**Table 1:** Variable inclusion in ML algorithms.

**Variables**	**Attribute and type**	**Details**
Gender	Categorical variable	Female (0), male (1)
Age	Continuous variable	Age of subjects
Family gastric cancer	Categorical variable	Gastric cancer history of first-degree relatives; No (0), yes (1)
H. Pylori test	Categorical variable	H. pylori infection; Negative (–,0),positive (+,1)
Vegetable intake	Categorical variable	Regular (0), occasional (1)
Fruit intake	Categorical variable	Regular (0), occasional (1)
Protein intake	Categorical variable	Intake of milk or beans; Regular (0), occasional (1)
PGI	Continuous variable	Serum pepsinogen I
PGII	Continuous variable	Serum pepsinogen II
High-salt diet	Categorical variable	Salt ≤ 10 grams a day (0), Salt>10 grams a day (1)
PGR	Continuous variable	The ratio of pepsinogen i and pepsinogen ii
Smoking	Categorical variable	>1 cigarette a day for more than a year (1), else (0)
Alcohol	Categorical variable	At least once a day for more than a year (1), else (0)

This study conducted a novel machine learning algorithm, XGBoost, compared with three other machine learning algorithms. The research data were divided randomly into training and test sets with a ratio of 4:1 (2,904/726). GridSearch (Bao and Liu, 2006) was used to find the optimal parameters of the model. Evaluation indicators such as accuracy, area under the receiver operator characteristic curve (AUC), positive predictive value, and sensitivity were calculated to compare the performance of algorithms. The best machine learning algorithm with optimal parameters was obtained while the highest AUC in our test set.

### Ethics and consent

**Figure 2:**
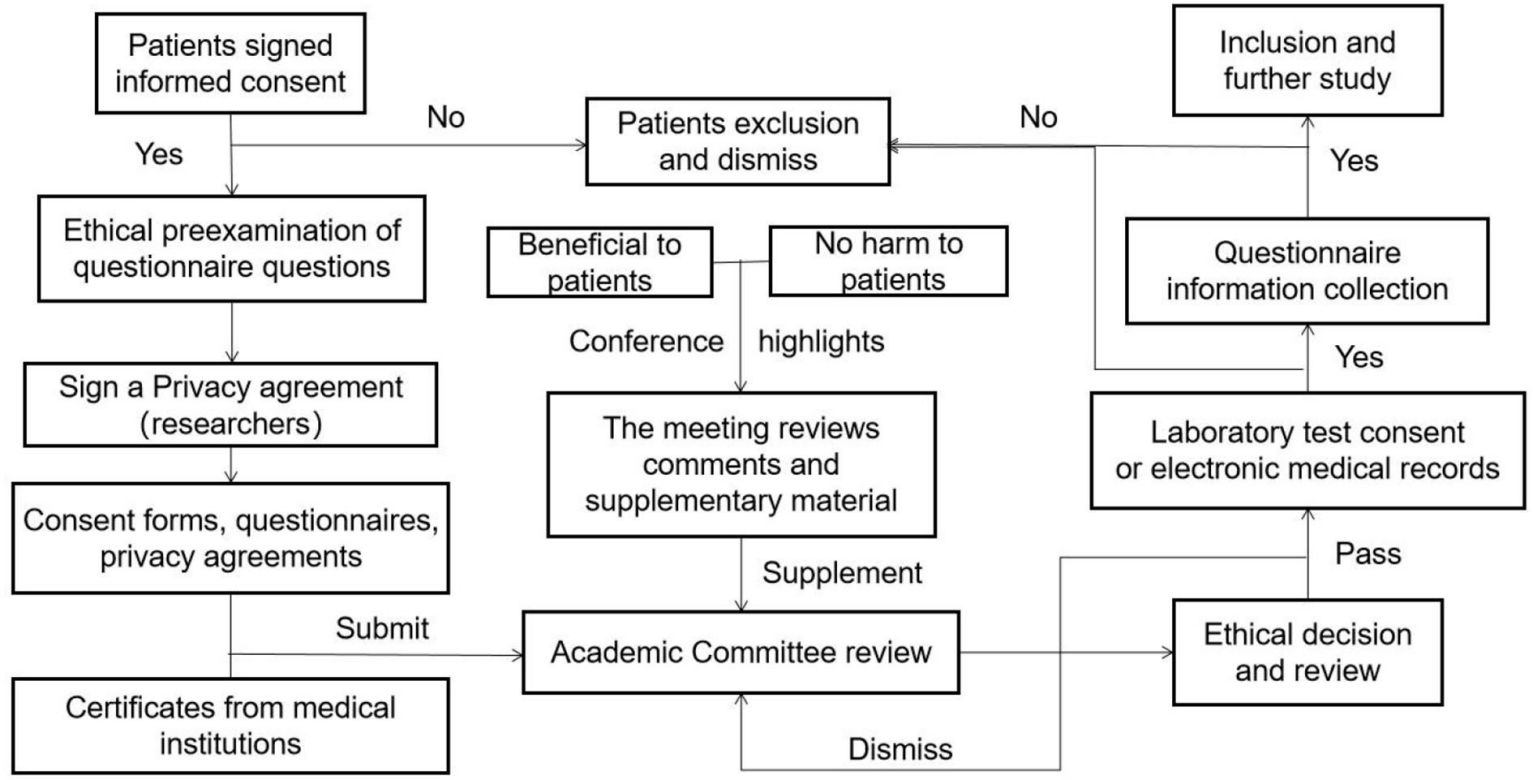
The ethical review process of subjects.

This study was approved and authorized by ethics approval. The ethical review process for the medical institution is shown in Figure 2, informed consent was obtained from all the patients before enrolment, and the use of information must be approved by the applicant or their guardian. The relevant laboratory data during the survey period were retrieved from the patient's electronic medical records.

### Study subjects

We conducted a retrospective study with 4,382 subjects from September 2017 to June 2020, and the details of the survey process were shown in Table 2.

**Table 2:** Details of annual survey subjects and information collection.

**Year**	**2017**	**2018**	**2019**	**2020**	**Amount**
**Survey samples**	859	1,251	1,287	985	4,382

**Figure 3:**
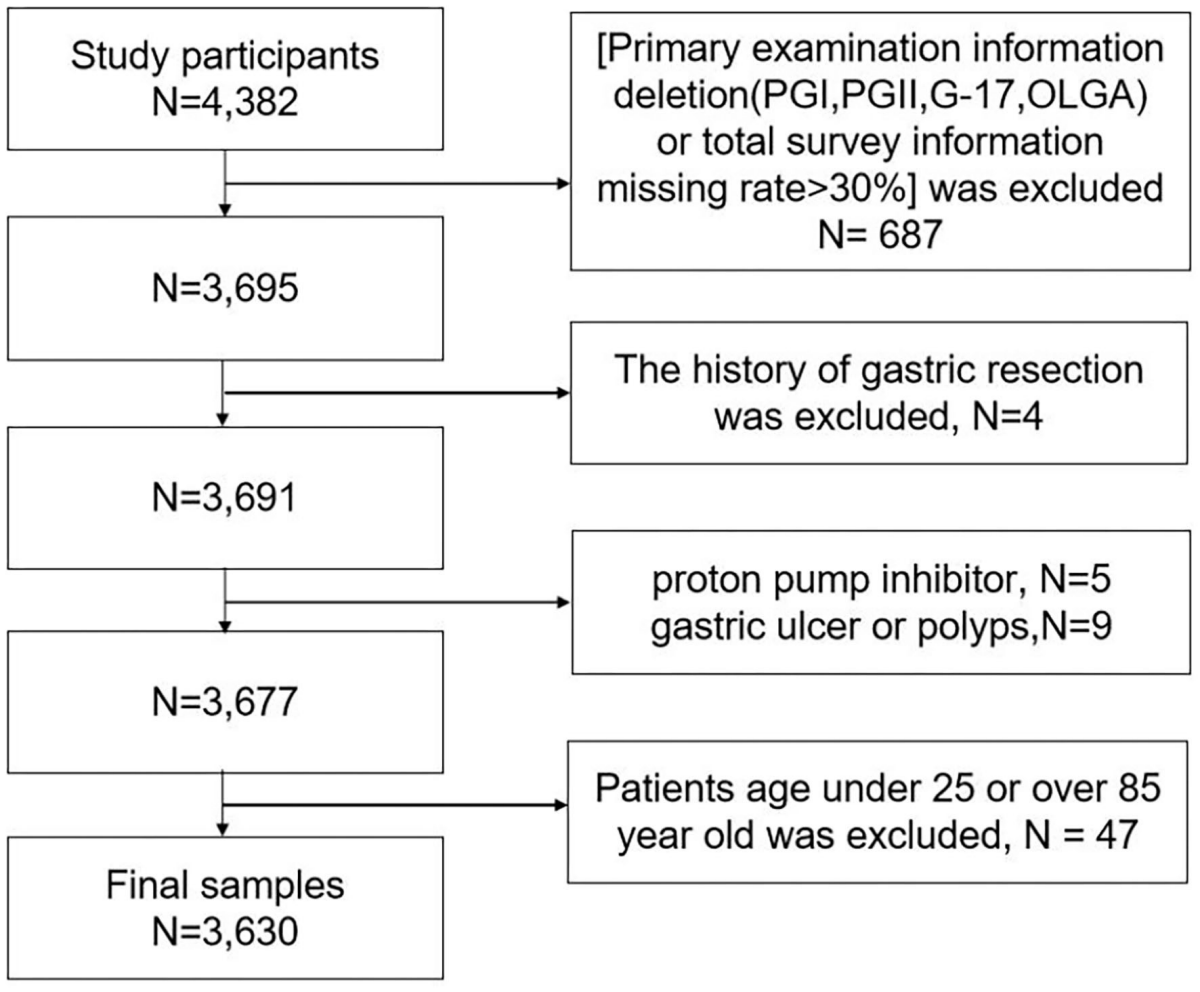
The process of inclusion and elimination.

The questionnaire consisted of baseline information (age, sex, and nationality); lifestyle (smoking consumption condition{at least a cigarette a day, > 1 year}, smokers were asked for the number of cigarettes and duration of smoking, drinking state{alcohol consumption at least twice a week, over a year}, and the drink frequency; dietary habits (the intake of salt more than 10 g a day as a high-salt diet, approximately assessment intake of daily oil, protein{bean, egg, milk, and meat}, spicy and sugar); green vegetables and fresh fruits {> 3 times a week}; The first-degree relatives gastric cancer history. Inclusion criteria during survey: (1) Guarantee a good compliance during the physical examination; (2) Age 25–85 years. And the exclusion criteria are as follows: (1) Refuse to sign informed consent; (2) History of esophageal, gastric polyp, and ulcer; (3) Existence of gastrointestinal warning symptoms such as gastrointestinal bleeding, persistent vomiting, anemia, abdominal mass, and dysphagia. (4) Severe mental illness affects normal investigation or compliance is poor, (5) Incomplete data (absence data of quantitative characteristics or individual total information missing rate>30%). In total, 3,630 subjects were enrolled in further study, the details of sample inclusion and elimination are shown in Figure 3.

### Clinical and biochemical data collection

Clinical data included H. pylori, PGI, and PGII. If these clinical indicators have been tested earlier, the relevant laboratory data during the survey period were retrieved from patient's electronic medical records. If there is no previous testing or record of clinical indicators, we would inform patients to sign informed consent forms and visit the nearest medical facility for examination, which included the serological markers and H. pylori (Ono et al., 2000).

### Statistical analyses

SPSS 19.0 software was used for statistical analysis. Quantitative variables were expressed by mean ± SD, a *T*-test was used to compare two continuous variable groups that follow a normal distribution, and a Chi-square test was used to compare the adoption rate or percentage of the categorical data. Variables with statistically significant differences in single factor analysis were included in multivariate analysis, OR-value and 95% CI were calculated to assess the related factors. *P* < 0.05 was considered as a statistically significant difference.

### Study outcome

According to the Operative Link on Gastritis Assessment (OLGA, which is a judgment indicator of the gastric mucosa atrophy degree in a patient by a professional physician and obtained from the laboratory data) standards and the Sydney level system (Dixon et al., 1996; Zhou et al., 2016), basic statistical information of subjects was compared among the NAG (non-atrophy group, OLGA-0 group), MAG (mild-moderate atrophy group, OLGA I-II group), SAG (severe-atrophy group, OLGA III-IV group) and GC (gastric cancer) groups. In the machine learning prediction process, we split the results into gastric cancer (+) and non-gastric cancer (-). We defined the NAG, MAG, and SAG groups combined as the non-gastric cancer group and compared them the gastric cancer (GC) group.

### Training algorithm

**Figure 4:**
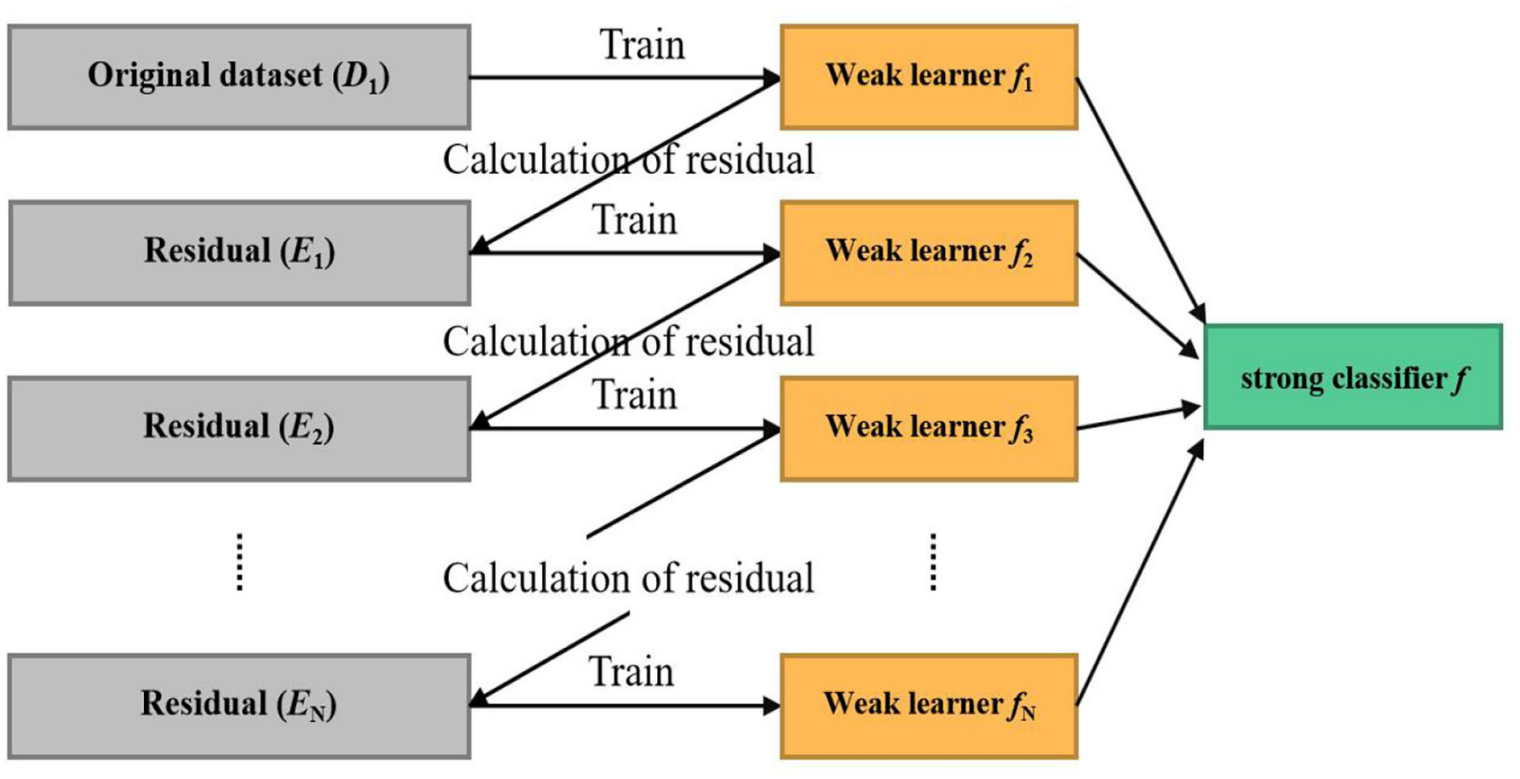
The framework of extreme gradient boosting algorithm.

Machine learning aims to make the learned functions apply well to the “new sample,” defining that the ability to use the intellectual process to a new sample is known as generalization ability. These algorithms attempt to excavate from many historical data implicit rules and are used to predict or classify. XGBoost (eXtreme Gradient Boosting) (Chen and Guestrin, 2016) was constructed with the Scikit-Learn package in Python. The architecture of the XGBoost is shown in Figure 4. XGBoost is an efficient algorithm based on the Gradient Boosted Decision Tree (GBDT) (Safavian and Landgrebe, 1991), which has attracted extensive attention because of its excellent learning capacity and efficient training speed. Compared with the GBDT, the proposed XGBoost algorithm mainly optimized objective function through the following three steps:

- The second order Taylor expansion removes constant and optimizes the loss function term.
- Regularization term expansion, removes constant term, and further optimizes regularization term.
- The final objective function is obtained by combining the first- and second-term coefficients.

### The algorithm principle of XGBoost

There is a training dataset {*D*_*T*_ = (*x*_1_, *y*_1_), (*x*_2_, *y*_2_), …, (*x*_*n*_, *y*_*n*_)}, parameter *x* means the input feature part of the training dataset, and parameter *y* represents the label (OLGA-I, II, III, and IV levels), sample (*x*_*i*_, *yi*) ranks *ith* in the training dataset, the theory was as follows.

1. First, initialized the model and get a general expression for the prediction:

$$\begin{array}{l} {ŷ}_{i}=\overset{K}{\underset{k=1}{\sum }}f_{k}\left(x_{i}\right) \end{array}$$

Where *f*_*k*_ *(x*_*i*_*)* is the initialized learner.

2. The overall objective function can be written as:

$$\begin{array}{l} \zeta \left(\Phi \right)=\underset{i}{\sum }l\left(y_{i},{ŷ}_{i}\right)+\underset{k}{\sum }\Omega \left(f_{k}\right) \end{array}$$

Where the loss *l*(*y*_*i*_, ŷ_*i*_) and the regularization term Ω(*f*):


$$\begin{array}{l} \Omega \left(f\right)=\gamma T+\frac{1}{2}\lambda {\left||w|\right|}^{2} \end{array}$$

3. The quadratic Taylor expansion can be obtained by substituting it into the objective function:

$$\begin{array}{l} L^{\left(t\right)}=\overset{n}{\underset{i=1}{\sum }}\left[l\left(y_{i},{ŷ}_{i}^{\left(t-1\right)}\right)+g_{i}f_{t}\left(x_{i}\right)+\frac{1}{2}h_{i}{f_{t}}^{2}\left(x_{i}\right)\right]+\underset{k}{\sum }\Omega \left(f_{k}\right) \end{array}$$

Where *g*_*i*_ and *h*_*i*_ are, respectively, the first and second derivatives of $l\left(y_{i},{{ŷ}_{i}}^{\left(t-1\right)}\right)$, removing constant term $l\left(y_{i},{{ŷ}_{i}}^{\left(t-1\right)}\right)$, given the leaf node *I*_*j*_ = {*i*|*q*(*x*_*i*_) = *j*}, mapping the sample to the leaf space (*T*) in order to get a quadratic function of ω_*j*_, obtained the final objective function:


$$\begin{array}{l} {\tilde{L}}^{\left(t\right)}=\overset{n}{\underset{i=1}{\sum }}\left[g_{i}f_{t}\left(x_{i}\right)+\frac{1}{2}h_{i}{f_{t}}^{2}\left(x_{i}\right)\right]+\left(\gamma T+\frac{1}{2}\lambda \overset{T}{\underset{j=1}{\sum }}\omega_{j}^{2}\right)\\ \;=\overset{T}{\underset{j=1}{\sum }}\left[\left(\underset{i\in Ij}{\sum }g_{i}\right)\omega_{j}+\frac{1}{2}\left(\underset{i\in Ij}{\sum }h_{i}+\lambda \right)\omega_{j}^{2}\right]+\gamma T\\ \;=\overset{T}{\underset{j=1}{\sum }}\left[G_{j}\omega_{j}+\frac{1}{2}\left(H_{j}+\lambda \right)\omega_{j}^{2}\right]+\gamma T \end{array}$$

4. Determined the optimal segmentation point and the optimal objective value:

$$\begin{array}{l} \omega_{j}^{*}=-\frac{G_{j}}{H_{j}+\lambda }\\ {\tilde{L}}^{\left(t\right)}=-\frac{1}{2}\overset{T}{\underset{j=1}{\sum }}\frac{G_{j}^{2}}{H_{j}+\lambda }+\gamma T \end{array}$$

5. The Segmentation Point With Maximum Gain Was Found by *G*_*L*_*, H*_*L*_*, G*_*R*_, and *H*_*R*_, and the Output Is the Split With max Score:

$$\begin{array}{l} Score=Max\left(Score,\frac{G_{L}^{2}}{H_{L}+\lambda }+\frac{G_{R}^{2}}{H_{R}+\lambda }-\frac{G^{2}}{H_{}+\lambda }\right) \end{array}$$

Where *G* means the sum of the second derivatives of all the samples, and *H* means the sum of the first derivatives of all the samples, *G* = *G*_*L*_ + *G*_*R*_ and *H* = *H*_*L*_ + *H*_*R*_.

### Tuning parameters of XGBoost

The parameters of XGBoost consist mainly of general parameter, booster parameter, and objective parameter. We set the general parameters (nthread and silent) to a default value, the objective parameter as “Linear,” and the reg_lambda parameter to 0.4 to prevent model overfitting. The booster parameters were tuned as follows:

- Booster parameters adopted default values of which the max_depth=6 and min_child_weight=1. A GridSearch was used to find optimum n_estimator and learning_rate to search the maximum number of iterations and appropriate learning efficiency.
- The n_estimator search range was set from 0 to 1,000. The step was 100, the initial learning rate was 0.01, step of 0.02. We selected the optimal parameters with the highest accuracy, and it was found that the n_estimator was 100 and the learning rate was 0.31 works optimal.
- Then performed, a further precise search for these parameters, n_estimator was set from 0 to 100 and the step was 1, the initial learning rate was 0.29, step=0.005. While n_estimator=18 and learning_rate=0.305, XGBoost algorithm reached higher accuracy.
- N_estimator was set to 18, and the learning rate was 0.305, fixed at this stage. GridSearch was be used to select the optimum max_depth and sub_sample parameters to find the maximum depth of the tree. The proportion of random sampling, a finite set for grid search max_depth was {3,4,5,6,7,8,9,10}, the search range of sub_sample was set from 0.55 to 1, the optimal parameters of max_depth and sub_sample were 5 and 0.9, which won the highest accuracy and the parameter of optimal performance was displayed in Table 3.

**Table 3:** Parameter presupposition of XGBoost algorithm.

**Parameters**	**Values**	**Parameters**	**Values**
learning_rate	0.305	n_estimator	18
max_depth	5	min_child_weight	1
sub_sample	0.9	n_ jobs	48
reg_lambda	0.4	reg_alpha	0

### Testing algorithm

After completing the training of the gastric cancer diagnostic model training, a test set consisting of 726 subjects was applied to evaluate the algorithm's performance. The receiver operating characteristics curve (ROC) was plotted by a threshold of 0.5. In total, four common effect indicators were used to evaluate the performance of different algorithms in GC diagnostic tasks:


$$\begin{array}{l} \begin{array}{c} Acc=\frac{TP+TN}{TP+TN+FP+FN}\\ Sens=\frac{TP}{TP+FN}\\ Spec=\frac{TN}{TN+FP}\\ PPv=\frac{TP}{TP+FP} \end{array} \end{array}$$

Where,

–True Positive (TP): Correctly predicted as positive.

–True Negative (TN): Correctly predicted as negative.

–False Positive (FP): Incorrectly predicted as positive.

–False Negative (FN): Incorrectly predicted as negative.

## Result

### Statistical analyses of baseline information

As shown in Table 4, 3,630 subjects were finally enrolled in this study. Of these subjects, 56.1% (2,034/3,630) were men and 43.9% (1,596/3,630) women, with an average age of 54.76±11.24 (years) and sex ratio of 1.276:1 (men/women). There were 2,983 samples in the NAG group (82.2%), 446 in the MAG group (12.3%), 77 in the SAG group (2.1%), and 124 in the GC group (3.4%), while the infection rate of H. pylori was 68.9%. The pepsinogen I (PGI) levels were statistically significantly lower in men than women in the OLGA-II and OLGA-III groups (*p* < 0.05), and the levels of pepsinogen II (PGII) in the OLGA-IV group was statistical significantly higher than OLGA-I, OLGA-II, and OLGA-III groups (*p* < 0.05).

**Table 4:** Characteristics of the subjects.

**Characteristics**	**Total**	**NAG group (*n* = 2,983)**	**AG/GC group (*****n*** = **647)**				**P^&^**
			**Total**	**MAG**	**SAG**	**GC**	
**Sex**							0.408
Male, *n* (%)	2,034	1,662 (55.7)	372 (57.5)	251 (56.3)	46 (59.7)	75 (60.5)	
Female, *n* (%)	1,596	1,321 (44.3)	275 (42.5)	195 (43.7)	31 (40.3)	49 (39.5)	
**Age (years)**							<0.001
<45	760	659 (22.1)ac	101 (15.6)	63 (14.1)b	20 (26.0)a	18 (14.5)	
45-65	2252	1863 (62.5)^a^	389 (60.1)	298 (66.8)^a^	46 (59.7)^a^	45 (36.3)	
>65	618	461 (15.5)^ac^	157 (24.3)	85 (19.1)^ab^	11 (14.3)^a^	61 (49.2)	
**Family history**							0.024
Yes, *n* (%)	433	339 (11.4)^c^	94 (14.5)	68 (15.2)	11 (14.3)	15 (12.1)	
No, *n* (%)	3,197	2,644 (88.6)	553 (85.5)	378 (84.8)	66 (85.7)	109 (87.9)	
**Vegetable**							<0.001
Occasional, *n* (%)	1,019	843 (28.3)^ab^	176 (27.2)	120 (26.9)	27 (35.1)^a^	29 (23.4)	
Regular, ±*n* (%)	2,611	2,140 (71.7)^b^	471 (72.8)	326 (73.1)^b^	50 (64.9)^a^	95 (76.6)	
**Fruits**							0.011
Occasional, *n* (%)	2,508	2,088 (70.0)^ac^	420 (64.9)	266 (59.6)^a^	50 (64.9)^a^	114 (91.9)	
Regular, *n* (%)	1,122	895 (30.0)^ac^	227 (35.1)	180 (40.4)^a^	27 (35.1)^a^	10 (8.1)	
**Alcohol**							0.023
Yes, *n* (%)	2,805	2,301 (77.1)	504 (77.9)	349 (78.3)	57 (74.0)	98 (79.0)	
No, *n* (%)	825	682 (22.9)	143 (22.1)	97 (21.7)^b^	20 (26.0)	26 (21.0)	
**Smoking**							0.025
Yes, *n* (%)	2,756	2,266 (76.0)	490 (75.7)	328 (73.5)	64 (83.1)	98 (79.0)	
No, *n* (%)	874	717 (24.0)^b^	157 (24.3)	118 (26.5)^b^	13 (16.9)^a^	26 (21.0)	
**H. pylori**							<0.001
Positive, *n* (%)	2,501	1,930 (64.7)^abc^	571 (88.3)	376 (84.3)	71 (92.2)	124 (100)	
Negative, *n* (%)	1,129	1,053 (35.3)^abc^	76 (11.7)	70 (15.7)^ab^	6 (7.8)^a^	0 (0)	
**Milk**							0.251
Regular, *n* (%)	500	420 (14.1)	80 (12.4)	52 (11.7)^b^	12 (15.6)	16 (12.9)	
Occasional, *n* (%)	3,130	2,563 (85.9)	567 (87.6)	394 (88.3)	65 (84.4)	108 (87.1)	
**Bean**							0.006
Regular, *n* (%)	1,094	928 (31.1)^ab^	166 (25.7)	114 (25.6)^ab^	53 (68.8)	96 (77.4)	
Occasional, *n* (%)	2,536	2,055 (68.9)^ab^	481 (74.3)	332 (74.4)^ab^	24 (31.2)	28 (22.6)	
**High-salt diet**							0.024
Yes, *n* (%)	381	329 (11.0)^bc^	52 (8.0)	30 (6.7)^a^	5 (6.5)^a^	17 (13.7)	
No, *n* (%)	3,249	2,654 (89.0)	595 (92.0)	461 (93.3)	72 (93.5)	107 (86.3)	
PGI(ng/ml), mean (SD)	118.9 (98.0)	119.5^ab^ (76.5)	115.8 (76.0)	110.1^a^ (74.9)	102.7^a^ (61.8)	144.6 (81.3)	0.261
PGII(ng/ml), mean (SD)	11.5 (9.7)	11.3^a^ (9.5)	12.3 (10.4)	11.7 (9.6)	10.5^a^ (8.5)	15.1 (13.5)	0.022
PGR, mean (SD)	13.6 (13.5)	13.8 (12.7)	13.0 (16.9)	12.9 (19.6)	12.8 (8.9)	13.2 (7.5)	0.201

*GC, gastric cancer; SAG, severe atrophic; MAG, mild–moderate atrophic; NAG, non-atrophic*.

*Regular (≥ 3 times/week) and occasional (<3 times/week)*.

a *(MAG, SAG, and NAG) group compared with the GC group, p < 0.05*.

b *(MAG and NAG) group compared with the SAG group, p < 0.05*.

c *NAG group compared with the MAG group, p < 0.05*.

*P^&^ Total of AG/GC group vs. NAG group (p-value)*.

We performed multivariate analysis by selecting variables (*p* < 0.05) in Table 4 and found statistically significant differences in age, family history, vegetables, fruits, alcohol, smoking, high salt diet, H. pylori, and PGII (Table 5).

**Table 5:** Multivariate logistics regression analysis of influencing factors of gastric cancer.

**Characteristic**		**OR**	**95%CI**	***P* value**	**β**
Age(years)	<45	Reference			
	45–65	3.172	1.786–5.629	0.379	1.154
	>65	4.199	2.716–6.495	0.043	1.435
Family History	No, *n* (%)	Reference			
	Yes, *n* (%)	1.118	0.605–2.067	0.026	0.111
Vegetable	Occasional,n(%)	Reference			
	Regular, *n* (%)	0.384	0.255–0.583	0.031	−0.959
Fruits	Occasional,n(%)	Reference			
	Regular, *n* (%)	0.156	0.113–0.287	<0.01	−1.873
Alcohol	No, *n* (%)	Reference			
	Yes, *n* (%)	2.951	2.398–4.650	<0.01	1.082
Smoking	No, *n* (%)	Reference			
	Yes, *n* (%)	1.547	0.840-3.791	0.038	0.438
H. pylori	Negative, *n* (%)	Reference			
	Positive, *n* (%)	4.039	2.641–6.207	<0.01	1.396
PGII(ng/ml)	<9.2	Reference			
	≥9.2	1.758	1.324–4.016	<0.01	0.564

The model was constructed according to the beta coefficients obtained for the independent characteristics. The clinical characteristics of the training and test dataset are shown in Table 6.

**Table 6:** Clinicopathologic characteristics of subjects in the training and test dataset.

**Characteristic**		**Training dataset (*n* = 2,904,%)**	**Test dataset (*n* = 726,%)**
Age (years)	<45	657 (22.6%)	103 (14.2%)
	45–65	1,713 (59.0%)	539 (74.2%)
	>65	534 (18.4%)	84 (11.6%)
Family History	No, *n* (%)	351 (12.1%)	82 (11.3%)
	Yes, *n* (%)	2,553 (87.9%)	644 (88.7%)
Vegetable	Occasional, *n* (%)	817 (28.1%)	202 (27.8%)
	Regular, *n* (%)	2,087 (71.9%)	524 (72.2%)
Fruits	Occasional, *n* (%)	2,064 (71.1%)	444 (61.2%)
	Regular, *n* (%)	840 (28.9%)	282 (38.8%)
Alcohol	No, *n* (%)	663 (22.8%)	162 (22.3%)
	Yes, *n* (%)	2,241 (77.2%)	564 (77.7%)
Smoking	No, *n* (%)	703 (24.2%)	171 (23.6%)
	Yes, *n* (%)	2,201 (75.8%)	555 (76.4%)
H. pylori	Negative, *n* (%)	913 (31.4%)	216 (29.8%)
	Positive, *n* (%)	1,991 (68.6%)	510 (70.2%)
PGII (ng/ml)	<9.2	1,328 (45.7%)	323 (43.6%)
	≥9.2	1,576 (54.3%)	403 (56.4%)
Gastric cancer	No, *n* (%)	2,805 (96.6%)	701 (96.5%)
	Yes, *n* (%)	99 (3.4%)	25 (3.5%)

### Performance of machine learning

**Figure 5:**
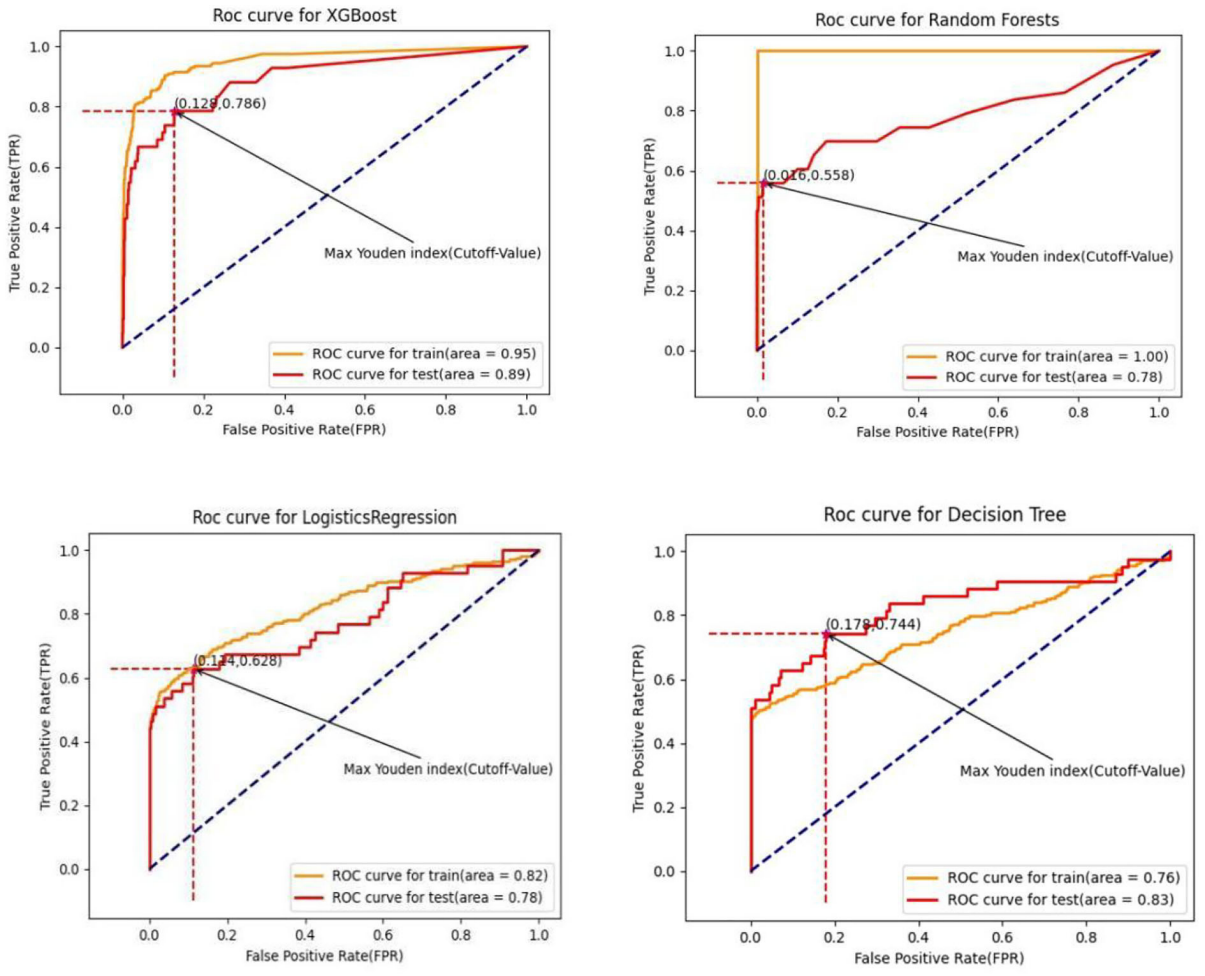
Receiver operating curve for algorithms.

Summarized evaluation results (test set for prediction) of the XGBoost algorithm and the other three machine learning algorithms are presented in Table 7. The corresponding training and test sets receiver operating characteristics curves of binary classifications (non-cancer as negative GC group and gastric cancer as positive GC group) were shown in Figure 5. The extreme gradient boosting algorithm achieved the optimal effect with an AUC of 0.891 (95% CI: 0.875–0.907), with a sensitivity of 0.787, specificity of 0.769, and positive predictive value of 0.738 at the operating point determined by the maximum Youden Index (Youden, 1950). The XGboost algorithm achieved significantly higher both AUC (0.891) and accuracy (0.857) compared with the other three machine learning algorithms (all *P* < 0.01). Compared with the random forest and logistic regression algorithms, the mean value of AUC in the decision tree algorithm was significantly different (*P* < 0.01), while no significant difference in AUC between random forest and logistic regression (*P* = 0.17).

**Table 7:** Performance among algorithms on the diagnosis of gastric cancer.

**Algorithm**	**AUC mean (SD)**	**Accuracy mean (SD)**	**Sensitivity mean (SD)**	**Specificity mean (SD)**	**PPV mean (SD)**
XGBoost	0.896 (0.005)	0.857 (0.008)	0.787 (0.010)	0.769 (0.011)	0.738 (0.009)
Random forest	0.786 (0.019)	0.724 (0.015)	0.559 (0.003)	0.983 (0.010)	0.663 (0.011)
Logistic regression	0.782 (0.017)	0.715 (0.010)	0.628 (0.019)	0.885 (0.013)	0.531 (0.004)
Decision Tree	0.833 (0.018)	0.784 (0.021)	0.745 (0.010)	0.821 (0.015)	0.689 (0.006)

*AUC, area under the receiver operating characteristic curve; SD, standard deviation; PPV, positive predictive value; XGBoost: extreme gradient boosting*.

The algorithm of the decision tree had the highest score of 0.566 in terms of the maximum Youden Index (sensitivity + specificity-1) among four algorithms, followed by XGBoost (0.556), logistic regression (0.542), and random forest (0.513) algorithm. Interestingly, the XGBoost algorithm performed the least specificity but the optimal sensitivity compared with the other three (all *P* < 0.01). Discrimination of the mean sensitivity between the XGBoost algorithm and the second highest one was 4.2%. In comparison, the discrimination of the mean positive predictive value of the XGBoost algorithm and the second one was 4.9%. Compared with XGBoost (73.8%), random forest (66.3%), and decision tree (68.9%), a baseline comparison logistic regression had the lowest PPV of 53.1%.

### Feature importance of variables among algorithms

**Figure 6:**
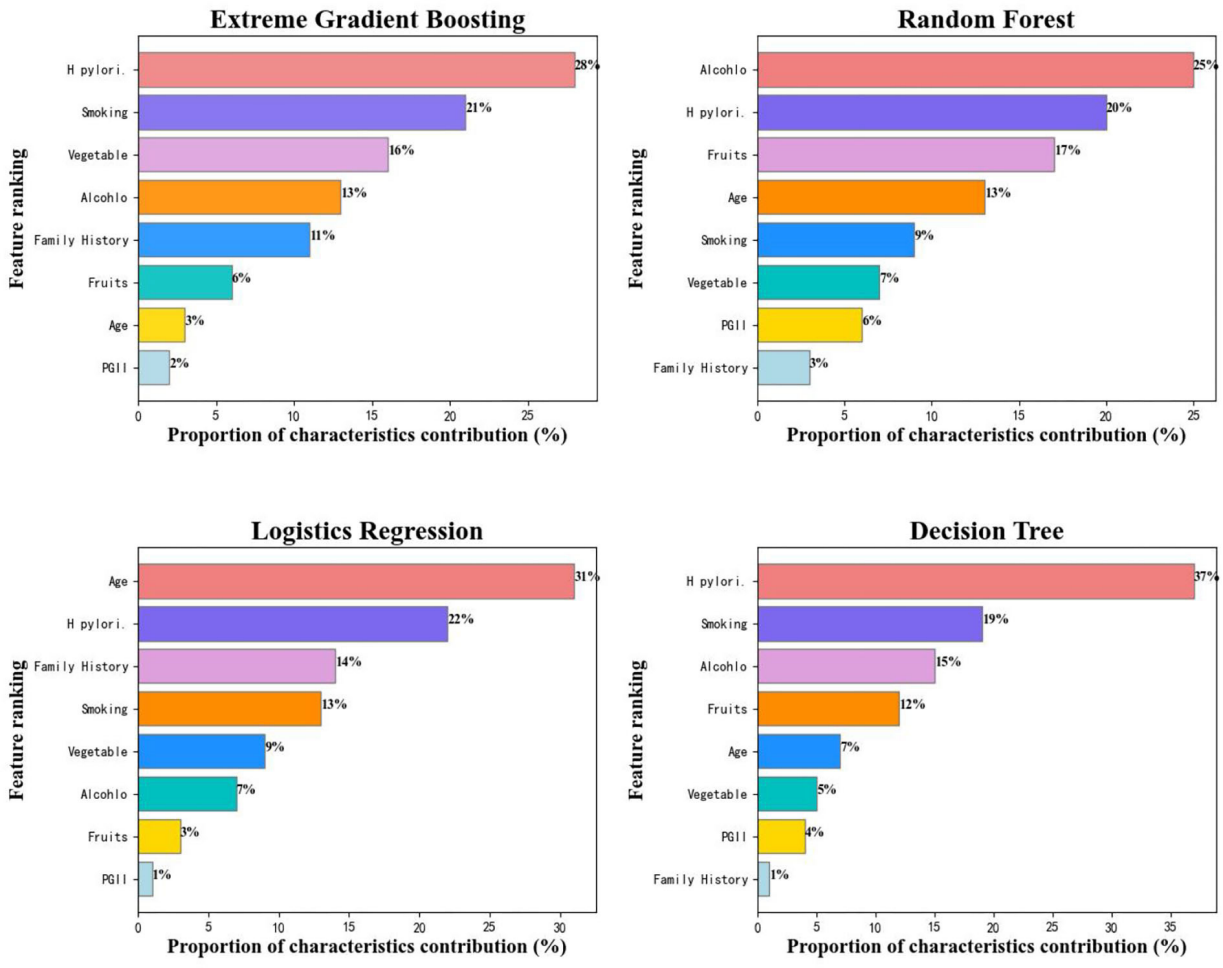
Characteristics ranking among different algorithms.

We further analyzed the feature importance of these eight variables among the four machine learning algorithms by calculating the feature score (*F*-score) of each variable for the predicted results, the higher the feature score is, the more contribution a variable makes to the detective of gastric cancer, and the relative contribution of each characteristic vector for mean AUC was quantified by the normalization of *F*-score. Figure 6 presented the variable rankings among different algorithms, although some discrepancies did exist, XGBoost and decision tree were very similar in variable rankings. Of these four algorithms, H. pylori, smoking, and age were considered as top-ranked variables while PGII had the least contribution to gastric cancer prediction. Compared with the other three algorithms, logistics regression model paid more attention to age (31%) and family history (14%) variables, ranked 1st and 3^rd^, respectively, while the smoking variable had a smaller influence on accurate prediction of the gastric cancer status (ranked 4th). We selected the XGBoost model (performed optimal in test set) for further analysis, and found that H. pylori was the most reliable variable, closely followed by smoking, age, vegetable intake, alcohol consumption, family history of GC, fruits, and PGII, which is similar to the previous study (Bornschein et al., 2012; Wang et al., 2014). Whereas further studies are required to explain the effectiveness of PGII in the decision tree-based algorithm gastric cancer prediction model.

## Discussion

The majority of patients with early GC are curable by appropriate treatments. With the progress made in radiotherapy, chemotherapy, and endoscopic resection, the 5-year survival rate of early GC can reach at least 95% (Song et al., 2017; Tan, 2019). Hence, early diagnosis plays a vital role in reducing the fatality rate (Karimi et al., 2014). Stomach endoscopic visualization is the only method available currently to detect gastric neoplasia and its precursors (Fernandes and Devis, 1994). However, despite the growing body of evidence on the practicability of endoscopic diagnostic for GC, the evidence remains poor, for it is based on studies other than randomized controlled trials (Hamashima, 2016). In addition, endoscopic diagnosis has the limitation of infection, invasiveness, and expensiveness. Thus, there is a crying demand to develop a safe, non-invasive, and cheap gastric cancer prediction diagnostic method with high accuracy for the early GC stage population.

This study found that age, family history, smoking, alcohol, fruits, vegetables, H. pylori infection, and PGII(ng/ml) were statistically significant factors for GC diagnosis. The multiple logistic regression analysis (Table 4) showed that age was one of the risk factors for the AG/GC group, especially subjects older than 65 years (OR = 4.20). Other risk factors included family history (OR = 1.12), alcohol (OR = 2.95), smoking (OR = 1.55), H. pylori infection (OR = 4.04), and PGII (OR = 1.76), while the intake of fruit (OR = 0.16) and vegetable (OR = 0.38) were two potential protective factors for the gastric carcinoma. A 20-year prospective cohort study in a Japanese population reported that the combination of serum pepsinogens (PGs) levels and H. pylori antibody was a powerful indicator of GC risk (Ikeda et al., 2016). Kikuchi et al. (1994) found serum pepsinogens could reflect the morphology and function of the gastric mucosa. Combining high-pepsinogen II levels with low-pepsinogen I/II ratios may be an efficient diagnostic method for gastric cancer in populations at increased risk. Shin et al. (2010) found that individuals with one first-degree family member with GC (OR = 2.7, 95% CI: 1.7–4.3) had a higher risk of gastric cancer. Furthermore, individuals with at least two first-degree family members with gastric cancer (OR = 9.6, 95%CI: 1.2–73.4) were at higher risk than single first-degree relatives. A meta-analysis study (Poorolajal et al., 2020) consisting of 232 studies demonstrated that H. pylori infection (OR = 2.65, 95%CI: 2.23–3.14) and current smoking (OR = 1.61, 95%CI: 1.49–1.75) were the first and second most important risk factors for gastric cancer, while fresh fruit (OR = 0.48, 95%CI: 0.37–0.63) and green vegetable (OR = 0.62, 95%CI: 0.49–0.79) intake were the top one and top two protective factors for GC. Our findings were consistent with previous reports.

Some researchers use tissue biomarkers (TFF2, mir-124a-3p, etc.), innovative blood biomarkers, and other low-cost markers (CEA, CA19-9, MUC16, etc.) to predict and evaluate the risk of gastric cancer. The comparison between other studies and our research results is shown in Table 8. For example, Kuo et al. (2017) found that serum TFF2 levels were associated with the degree of SPEM and the risk of GC. However, tissue biomarkers have the disadvantages of being invasive, the risk of adverse events (stress reaction), and the performance depend to some extent on the sampling site. Innovative blood biomarkers such as cell-free DNA and RNA (miR-25, miR-486-5p, etc.) achieve high accuracy in early diagnosis and can be obtained through minimally invasive methods. Zhu et al. (2014) found that a combination of plasma miR-92a, miR-16, and miR-25 indicated as a potential biomarker for diagnosing non-cardia gastric cancer, with an AUC of 0.812. Liu et al. (2011) showed that the performance of machine learning using plasma miR-20a, miR-1, miR-423-5p, and miR-34 reached an AUC of 0.867. Unfortunately, innovative blood biomarkers are not available in many medical institutions due to complexity and expensiveness. Non-invasive gastric cancer markers researcher Zhu et al. (2020) proposed a gastric cancer diagnosis method using the GBDT algorithm based on subjects' non-invasive characteristics (CEA, CA19-9, etc.), with an accuracy of 0.83. Nevertheless, conventional serum tumor biomarkers such as carcinoembryonic and carbohydrate antigen (CA) 19-9 are unsuitable for early gastric cancer diagnosis due to insufficient specificity and sensitivity.

**Table 8:** Comparison of different studies.

**Studies**	**Characteristics used for prediction**	**Weakness**	**Accuracy,%**
Kuo et al. (2017)	serum TFF2, serumTFF3, etc.	Invasive (bleeding, stress reaction, etc.)	73.4
Zhu et al. (2014)	miR-(16, 25, 92a, etc.)	complexity and expensiveness	78.2
Liu et al. (2011)	miR-(20a, 34, 423-5p, etc.)		84.9
Zhu et al. (2020)	CEA, CA19-9, CA-125, NLR, Hb, Alb, etc.	unsuitable for early diagnosis	83.1
Ours	PGII, H. pylori, smoking, etc.	Subjectivity	85.7^*^

*TFF, trefoil factor; CEA, carcinoma embryonic antigen; CA, carbohydrate antigen; NLR, neutrophil-lymphocyte ratio; Alb, albumin; Hb, hemoglobin; PGII, Pepsinogen II; H. pylori, Helicobacter pylori*.

* *The maximum accuracy obtained*.

The prevalence rate of gastric cancer is 3.4% (124/3,630) in this study, there is a problem of imbalance in training samples. We simulated 500 positive samples of gastric cancer by SMOTE algorithm (Blagus and Lusa, 2013), then set the scale_pos_weight parameter to 0.178 (positive sample/negative sample), the purpose is to over-sample few sample categories and under-sample the various sample categories to balance the learning degree of the two types of labels. In the unbalanced problem, the value of AUC has a higher priority than accuracy, and the positive predictive value is more relevant in a clinical setting because it assesses the probability that patients who test positive will develop the target cancer. The sensitivity of diagnostic tests is more important than specificity in the early diagnosis of cancer-related diseases (Pinsky, 2015). Whereas the low prevalence in the gastric cancer diagnostic environment resulted in a very high negative predictive value, which was useless in rare disease to assess the performance of the model for providing less information (Pinsky, 2015). Compared with the other three models, the XGBoost model performed optimal in sensitivity (78.7%) and positive predictive value (73.8%). Logistic regression (Marill, 2004) is commonly applied to medical statistical analysis of the influencing factors. The main reason logistic regression in terms of AUC and positive predictive value is lower than XBGoost is that the characteristics of logistic regression of each dimension are independent, so it only has the ability to the existing feature space segmentation (Hou et al., 2020). Moreover, XGBoost does not raise the dimension of the feature space and seeks the split-point with the smallest residuals, so it will automatically seek other features that can minimize residuals under the current split subtree (Li et al., 2021), and in this manner, it will automatically have the performance of finding good feature combinations and give essential elements according to the residuals decrease. The decision tree is used usually in feature selection or classification problems with a significant difference in data attribute range and sparse data. It is widely used in financial sub-control (Nobre and Neves, 2019), medically assisted diagnosis, and other fields with satisfactory results. However, compared with the decision tree, the random forest has higher stability, the prediction results are obtained by referring to multiple decision trees, which reduces the influence of outliers. In this study, random forest and decision tree algorithms have obtained AUC of 0.79 and 0.83, respectively. The better effect of decision trees may be due to the inaccurate prediction of some decision trees due to the influence of outliers. Early studies have shown that XGBoost works better in low-dimensional data, XGBoost has better generalization ability and stability when dealing with unbalanced data sets. Small changes in hyperparameters of the random forest will affect almost all the decision trees and change the prediction results, while XGBoost pays more attention to functional space and can achieve stable and efficient prediction with very few parameters (Li et al., 2018). Random forest is an ensemble classifier method based on a decision tree with strong noise resistance and robustness. Compared with the XGBoost algorithm, the random forest performs better in high-dimensional data with more noise (Kong et al., 2017).

The most significant highlight of the current study is that this is the first study to develop a GC-predictive model based on lifestyle behavior and non-invasive characteristics using machine learning methods. In addition, the medical cost of this study is acceptable, and similar information of subjects is procurable in many digestive system departments of medical institutions. Nevertheless, limitations also existed in this study. A significant disadvantage of this study is that the sample size of gastric patients with gastric cancer is limited, leading to differences in the learning degree of different types of the training process so that the learning degree of patients with non-gastric cancer is easy to overfit but still under-fitting for patients with gastric cancer. Second, there would be some subjective bias involving the variable assignments and the survey of lifestyle behavior, which may influence the prediction results. In addition, a limitation of this approach was not the part of a clinical trial and cannot be used to diagnose gastric cancer clinically until further investigation and approval of authorities. At last, the results require further validation because of the relatively imbalanced small sample size.

## Conclusion

The study aimed at working out the problem that the prediction accuracy and the generalization performance of proposed methods are not satisfied in the field of predicting the risk of suffering from gastric cancer. The gastric cancer status of random samples was predicted based on the personal information, eating habits, family history of the included population surveyed, and several non-invasive biochemical data collections. Our research has shown that the novel machine-learning algorithm XGBoost achieved better discriminatory precision in identifying individuals' gastric cancer status than the other three algorithms, which is the optimal choice for developing a GC diagnosis model using a combination of non-invasive clinical characteristics and lifestyle behaviors factors. We will better estimate the risk of potential patients with gastric cancer and provides new ideas for intelligent prevention of gastrointestinal diseases in the future.

## Data availability statement

The original contributions presented in the study are included in the article/supplementary material, further inquiries can be directed to the corresponding authors.

## Ethics statement

The studies involving human participants were reviewed and approved by Nos. 2015-082 and 2019-132, authorized by the Second Affiliated Hospital of Zhejiang University. The patients/participants provided their written informed consent to participate in this study.

## Author contributions

SJ, HG, JS, and JW: conceptualization. JS, SJ, and HG: methodology. JS and SJ: software. JS and HG: validation. SJ and HG: formal analysis and visualization. JW: resources and supervision. YT and SJ: data curation. SJ: writing—original draft preparation. SJ and JW: writing—review and editing. YT and JW: project administration and funding acquisition. All authors have read and agreed to the published version of the manuscript.

## Conflict of interest

The authors declare that the research was conducted in the absence of any commercial or financial relationships that could be construed as a potential conflict of interest.

## Publisher's note

All claims expressed in this article are solely those of the authors and do not necessarily represent those of their affiliated organizations, or those of the publisher, the editors and the reviewers. Any product that may be evaluated in this article, or claim that may be made by its manufacturer, is not guaranteed or endorsed by the publisher.
